# Generation and Analysis of Expressed Sequence Tags from *Chimonanthus praecox* (Wintersweet) Flowers for Discovering Stress-Responsive and Floral Development-Related Genes

**DOI:** 10.1155/2012/134596

**Published:** 2012-03-21

**Authors:** Shunzhao Sui, Jianghui Luo, Jing Ma, Qinlong Zhu, Xinghua Lei, Mingyang Li

**Affiliations:** ^1^College of Horticulture and Landscape, Chongqing Engineering Research Center for Floriculture, Key Laboratory of Horticulture Science for Southern Mountainous Regions of Ministry of Education, Southwest University, Chongqing 400715, China; ^2^College of Life Science, South China Agricultural University, Guangzhou 510642, China; ^3^Department of Botony, Chongqing Agricultural School, Chongqing 401329, China

## Abstract

A complementary DNA library was constructed from the flowers of *Chimonanthus praecox*, an ornamental perennial shrub blossoming in winter in China. Eight hundred sixty-seven high-quality expressed sequence tag sequences with an average read length of 673.8 bp were acquired. A nonredundant set of 479 unigenes, including 94 contigs and 385 singletons, was identified after the expressed sequence tags were clustered and assembled. BLAST analysis against the nonredundant protein database and nonredundant nucleotide database revealed that 405 unigenes shared significant homology with known genes. The homologous unigenes were categorized according to Gene Ontology hierarchies (biological, cellular, and molecular). By BLAST analysis and Gene Ontology annotation, 95 unigenes involved in stress and defense and 19 unigenes related to floral development were identified based on existing knowledge. Twelve genes, of which 9 were annotated as “cold response,” were examined by real-time RT-PCR to understand the changes in expression patterns under cold stress and to validate the findings. Fourteen genes, including 11 genes related to floral development, were also detected by real-time RT-PCR to validate the expression patterns in the blooming process and in different tissues. This study provides a useful basis for the genomic analysis of *C. praecox*.

## 1. Introduction

*Chimonanthus praecox *(L.) Link, wintersweet, belongs to the Calycanthaceae family. It is a perennial deciduous shrub and blossoms in winter, from late November to March. Its unique flowering time and long blooming period make it one of most popular ornamental plants in China [1]. *C. praecox* is mainly a garden plant that also provides cut flowers. The flower is strongly fragrant and may be used as a source of essential oil, which has received much attention in New Zealand [2].* C. praecox* thrives in cold environments and blooms in low-temperature seasons with little rainfall. The plant is assumed to be rich in genes related to floral development and adversities, especially those responding to environmental stress factors. However, the molecular mechanism that regulates floral development and copes with stresses in *C. praecox* flowers remains unclear.

Expressed sequence tags (ESTs) have been proven to be an efficient and rapid means to identify novel genes (and proteins) induced by environmental changes or stresses [3–7]. Genes related to flower form, longevity, and scent from roses, *Phalaenopsis equestris*, and *Pandanus fascicularis* were identified by ESTs [8–10]. The present study used transcriptomic analysis of *C. praecox* flowers to identify novel genes induced by environmental changes or related to floral development and ultimately to better understand the physiological and genetic basis of cold acclimation in flowers of woody plants.

## 2. Materials and Methods

### 2.1. Complementary DNA (cDNA) Library Construction and Sequencing

**Figure 1:**
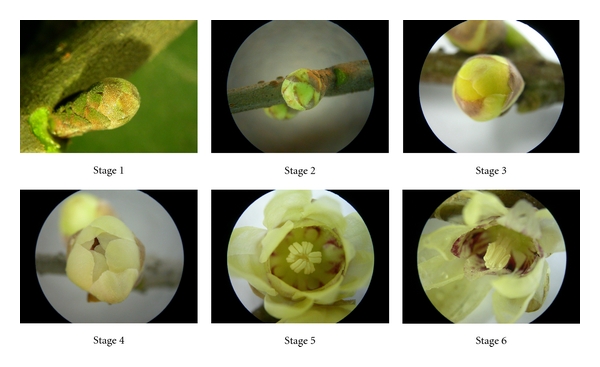
Stages of *C. praecox* blooming. Stage 1, sprout period: bud scales loosen; Stage 2, flower-bud period: flower buds turn green; Stage 3, display-petal period: flower buds enlarge and turn yellow; Stage 4, initiating bloom period: fragrance emerges; Stage 5, bloom period: flowers fully open with strong fragrance; Stage 6, wither period: petals begin withering.

The process of *C. praecox* blossoming includes the following: Stage 1, sprout period; Stage 2, flower-bud period; Stage 3, display-petal period; Stage 4, initiating bloom period; Stage 5, bloom period; Stage 6, wither period [11]. *C. praecox* flower buds or flowers at the six stages of development (Figure 1) were collected from the nursery at Southwest University, Chongqing, China, for cDNA library construction. The samples were immediately frozen with liquid nitrogen and then refrigerated at −80°C until RNA isolation. Total RNA samples were extracted from these flower buds or flowers using RNA isolation kits (W6771; Watson Biotechnologies Inc.), and RNA quality was detected by BioSpec-mini (Shimadzu). The final RNA sample for the cDNA library construction was bulked by pooling equal amounts of total RNA from each stage.

cDNA synthesis from the mixed total RNA and library construction through directional cloning of cDNAs into the *λ*TriplEx2 vector was performed using a SMART cDNA Library Construction Kit (K1051-1) according to the manufacturer's instructions. The size of the insert fragment and the recombinant rate were measured by PCR, as described by Gao and Hu [12], using a random selection of 50 clones. All clones were sequenced from the 5′ end using ABI 3700 (Applied Biosystems).

### 2.2. Sequence Clustering, Annotation, and Functional Categorization

Poor-quality sequences, sequences with less than 100 bases, and vector sequences were trimmed from the raw sequences using SeqMan II (DNASTAR, Inc., Madison, WI) and manually. The trimmed cDNA sequences were assembled into clusters using the assembly program within SeqMan II set to default parameters. The assembly parameters were set to require a minimum match of 80% over 12 bp to initiate the assembly process. A consensus sequence for unigenes was exported from SeqMan II.

Unigenes (contigs and singletons) were annotated using BLASTX against the NCBI nonredundant protein database with a cut-off *E* value of the best hit of ≤10^−5^ [13]. Sequences without a reliable match (>10^−5^) were subsequently compared with the NCBI nonredundant nucleotide database by performing BLASTN (score >100) for complementary annotation [14]. All well-annotated unigenes were then further classified and mapped to the three Gene Ontology (GO) categories (biological, cellular, and molecular) via AmiGO (http://amigo.geneontology.org/) [15].

### 2.3. Expression Analysis of Selected Genes Using Quantitative Real-Time RT-PCR

For real-time expression studies, *C. praecox *seeds were kept under a 16 h light/8 h dark cycle in a growth chamber at 25°C. Small seedlings were subsequently transferred to plastic pots containing a mixture of soilrite and vermiculite (1 : 1). The plantlets were raised to the sixth leaf stage and then subjected to cold treatment (4°C) for 15 min, 1 h, and 6 h. Control plantlets were maintained at 25°C. The tissues were harvested and snap-frozen in liquid nitrogen and kept at −80°C until further use.

The total RNA of flowers at the different developmental stages as well as floral organs dissected from Stage 4 flowers, roots, stems, and leaves were extracted with the above-mentioned method. Equal amounts of DNA-free RNA (5 *μ*g) from different tissues were reverse-transcribed using a PrimeScript RT Reagent Kit with gDNA Eraser (TaKaRa). The primers used for real-time PCR (Table 1) were designed by Primer Premier 5.0 (PREMIER Biosoft, USA). Real-time PCRs were performed in triplicate on 10 *μ*L reactions using 5 *μ*L of SsoFast EvaGreen Supermix (Bio-Rad), 0.5 *μ*L of each primer (final concentration of 500 nM), 3.5 *μ*L of water, and 0.5 *μ*L of cDNA template. Table 1 shows the primer pair-specific temperatures. PCR was carried out using a Bio-Rad CFX96 real-time system machine based on the manufacturer's instructions under the following conditions: denaturation at 95°C for 30 s; 40 cycles of 95°C for 5 s; 59°C for 5 s. Dissociation kinetics was performed using the real-time PCR system at the end of the experiment (60 to 95°C; continuous fluorescence measurement) to check the annealing specificity of the oligonucleotides. A comparative Ct (threshold cycles) method of relative quantification was used to analyze the real-time quantitative RT-PCR data using Bio-Rad CFX Manager Software Version 1.6. Actin and tubulin were used as housekeeping genes for the calculation of relative transcript abundance. The sizes of the amplified products were confirmed via gel electrophoresis. Negative controls with no templates were carried out concurrently.

## 3. Results

### 3.1. General Characteristics of the cDNA Library and the Ests

A cDNA library constructed from *C. praecox* flowers at different stages of development was used as a source of ESTs. The titers of the primary library and amplified library were 1.4×10^6^ and 1.0×10^10^ pfu/mL, respectively, with a recombinant rate of 96% for the original library. The sizes of the inserts ranged from 0.5 to 2.5 kb, and the average insert size was estimated to be 1.1 kb by PCR amplification of inserts from 50 randomly selected clones. These results indicate that our cDNA library was qualified.

**Figure 2:**
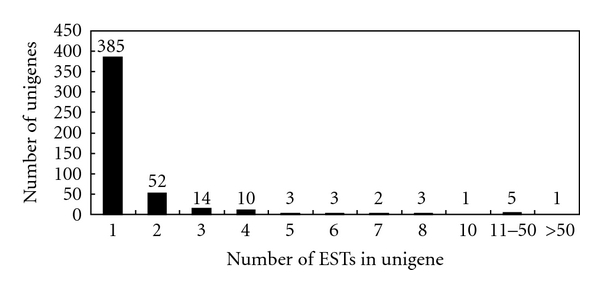
Distribution of *C. praecox* ESTs among unigenes.

In total, 896 random cDNA clones were successfully sequenced to generate ESTs. Trimming of the short sequences (<100 bp), vector sequences, and poor-quality sequences resulted in 867 high-quality ESTs, constituting a total of 584,201 bases in the *C. praecox* sequence. The average read length of these ESTs was 673.8 bp. All 867 sequences were deposited in GenBank under accession numbers DW222667 to DW223533. The clustering of ESTs generated 94 contigs (containing 2 or more ESTs) and 385 singletons (containing only 1 EST), yielding 479 unigenes. The redundancy of the library was calculated as 44.8% [(1 − Number of Unigenes/Number of ESTs) × 100%].  Figure 2 shows the distribution of ESTs in unigenes after clustering. Forty-two contigs had more than 2 ESTs, with the largest one containing 77 ESTs.

### 3.2. Functional Annotation and Classification of *C. praecox* Unigenes

The 479 unigenes were compared with the nonredundant protein and nucleotide sequences database in NCBI using BLAST. Four hundred five unique sequences, corresponding to 84.6% of all the unigenes, shared significant homology with sequences in the public databases. Of these, 266 were similar to genes of known functions, whereas 139 were similar to putative uncharacterized proteins (Table 2). The remaining 74 unigenes (15.4%) had only very weak or no matches and were considered as novel genes in *C. praecox* flowers.

Table 3 summarizes the highly expressed genes that contained more than 5 ESTs in one contig. The first and third most abundant ESTs were homologous to lipid transfer protein (LTP); the second most abundant ESTs encoded protein related to the adenine nucleotide translocator and then seed-specific protein, mannose-specific lectin, and LEA III protein (Table 3). These ESTs possibly corresponded to the most abundantly expressed genes in *C. praecox* flowers. Two hundred eighty-one ESTs were found to have more than five copies (Table 3), 385 had single copies of ESTs, and the remaining ESTs contained two to four copies in *C. praecox* flowers.

The database-matched 405 unigenes were found to have BLAST hits with 99 organisms, among which the highest number was from *Arabidopsis thaliana* (32.6%; 132 unigenes), followed by *Oryza sativa* (24.0%; 97 unigenes) and *Nicotiana tabacum* (4.2%; 17 unigenes) (Table 4). The remaining 67 unigenes (16.5%) had BLAST hits with a single organism only. The extensive distribution of the matched organisms may be attributed to the fact that the genome of *C. praecox* significantly differed from the genomes of model plants, as well as the fact that the relative plants' genomes have not yet been widely studied.

**Figure 3:**
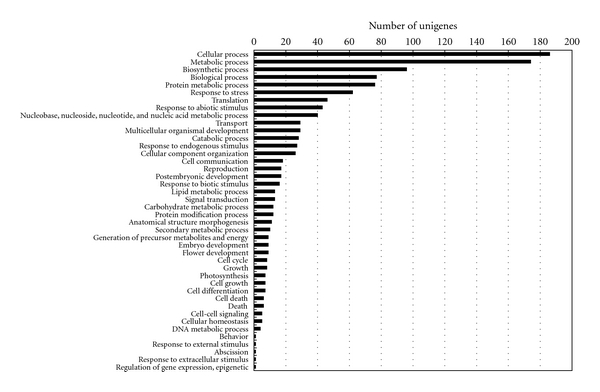
GO classification of the ESTs based on their biological functions in the *C. praecox* flower cDNA library.

**Figure 4:**
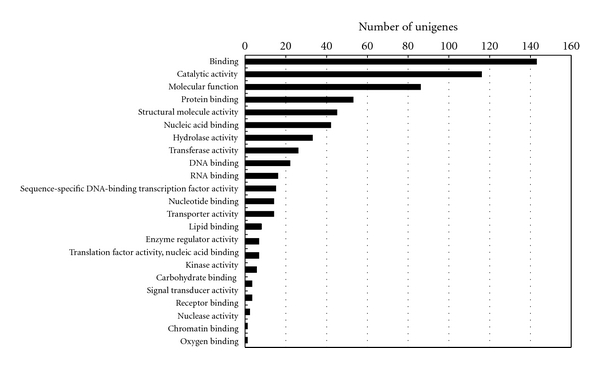
GO classification of the ESTs based on their molecular functions in the *C. praecox* flower cDNA library.

**Figure 5:**
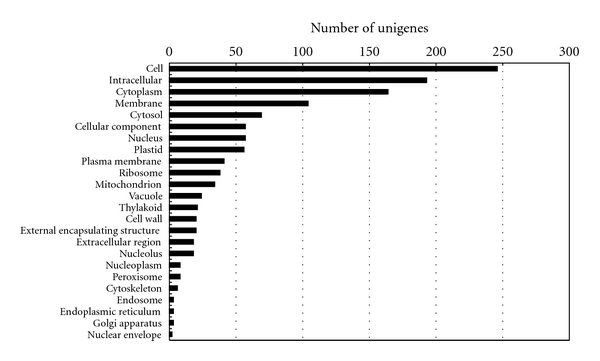
GO classification of the ESTs based on their cellular components in the *C. praecox* flower cDNA library.

The initial annotations were further simplified into plant-specific annotations (plant GO slim; http://amigo.geneontology.org/) to obtain additional insights into the putative functions of unigenes. Of the 479 *C. praecox* unigenes, 364 were assigned GO terms in any category (biological, cellular, and molecular). Figures 3, 4, and 5 classify the unigenes according to terms in the biological process ontology, molecular function ontology, and cellular component ontology, respectively.

### 3.3. Sequences Related to Stress and Defense

Among the ESTs that matched genes with known or putative functions, approximately 95 unigenes (291 ESTs) involved in stress and defense accounted for 19.8% (95/479) of all unigenes and 33.6% (291/867) of all ESTs.  Table 5 shows the nonredundant ESTs that share similarities with genes related to defense and stress response according to GO classifications and previously published data. As expected, the ESTs involved in stress and defense were highly abundant in the library because the *C. praecox* thrives and blossoms in winter, thereby confirming previous reports of related transcripts with higher levels of defense in developing flowers [16–20].

Twelve unigenes (22 ESTs) related to cold stress tolerance were found in the library; these were classified as “response to cold” according to GO terms [e.g., *β*-amylase, acyl-CoA-binding protein, 3-hydroxyisobutyryl-coenzyme A hydrolase, catalase, low-temperature and salt-responsive protein, glutathione *S*-transferase (GST), membrane channel protein, and abscisic-acid-induced protein] and “cold acclimation” (e.g., fatty acid biosynthesis 1 and WCOR413-like protein). In this class, the most abundant sequences encode GST. Five GSTs encoded by 16 ESTs were identified in the library. GST genes exhibited a diverse range of responses to jasmonates, salicylic acid, ethylene, as well as oxidative stress in *Arabidopsis* [21], and were induced by heavy metals and hypoxic stress in rice roots [22]. In the current study, however, not enough cold-related unigenes were obtained, as expected. Some unknown functional genes related to cold stress tolerance likely exist in this library.

Another class of genes involved in “response to absence of light,” “low light intensity,” and “response to red or blue light” according to GO terms was also represented in the library. Eight unigenes (10 ESTs) were identified, including acyl-CoA-binding protein, sadtomato protein, and catalase, among others.

Of the stress- and defense-related unigenes, 39 were possibly related to development. Nine types of LTPs or LTP precursors were encoded by the highest number of ESTs in this study (Table 5). Research has suggested different functions for LTPs in the physiology of plants, such as cutin synthesis, *β*-oxidation, somatic embryogenesis, pollen development, allergenics, plant signaling, and plant defense [23–25], but the true physiological role of LTPs in *C. praecox* flowers has yet to be determined. Fourteen ESTs encoding two kinds of LEA proteins, groups III and V, were identified. The presence of LEA proteins correlates well with freezing, water deficit, and salt stress [26–28], probably through the prevention of enzyme aggregation [29], and likely plays a similar role in *C. praecox*. Some other development-related unigenes were also found, such as MYB, calmodulin, actin, CCR4-associated factor, ubiquitin-conjugating enzyme E2, and ABC transporter, which are involved in transcription factor activity, signal transduction, cell structure, nucleotide metabolism, protein metabolic process, and transporter activity, respectively.

### 3.4. Sequences Related to Floral Development

Table 6 shows the 19 unigenes related to floral development. Five of these were homologous to MADS box transcription factor genes. Plant life critically depends on the function of MADS box genes encoding MADS domain transcription factors, which are present to a limited extent in nearly all major eukaryotic groups but constitute a large gene family in land plants [30]. 

MADS box genes control diverse developmental processes in flowering plants—ranging from root to flower and fruit development—and they especially control the processes of the transition from vegetative to reproductive development and establishment of floral organ identity [31]. The present study has also identified six unigenes related to secondary metabolism that are probably involved in floral color and fragrance.

The role of chilling temperature in dormancy in vegetative buds and induction of flowering has been investigated in many temperate-region species, particularly in *A. thaliana* and *Populus* spp. [32, 33]. The physiological processes of dormancy release and induction of flowering competence rely on longer-term chilling temperature and a period of vernalization, respectively [34, 35]. The current study has identified dormancy and vernalization-related genes; however, only one unigene (Cp82; *E*-value = 6.00*E* ^−15^) was annotated as a dormancy-related protein in the library The possible reason was only a small-scale sequencing in our study or the processes of dormancy release and induction of flowering competence in *C. praecox* only last a short period.

### 3.5. Expression Analysis of Cold-Responsive and Floral Development-Related Genes

**Figure 6:**
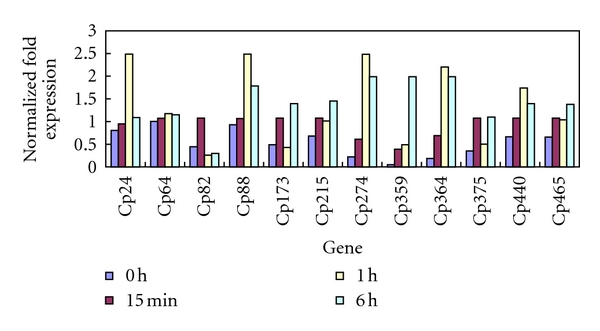
Expression analysis of 12 genes under cold stress (4°C).

Real-time RT-PCR was performed for 12 unigenes, including 9 selected from the GO Slim annotation belonging to “response to cold” (Cp88, Cp173, Cp215, Cp274, Cp359, Cp364, Cp375, Cp440, and Cp465), 1 annotated as a dormancy-related protein (Cp82), and 2 without functional annotation (Cp24 and Cp64), to analyze the changes in their expression due to cold stress (Figure 6). The data revealed that Cp82 responded to cold stress immediately after treatment and reached peak expression levels as early as 15 min. Cp24 (no annotation), Cp88 (membrane channel protein), Cp274 (GST), Cp364 (cold acclimation protein WCOR413-like protein), and Cp440 (multiple stress-responsive zinc-finger protein) were upregulated after 15 min of treatment and reached their expression peaks at 1 h. Cp173 (low-temperature and salt-responsive protein), Cp215 (abscisic acid-induced protein), Cp359 (1,4-alpha-glucan-maltohydrolase), Cp375 (3-oxoacyl-[acyl-carrier-protein] synthase), and Cp465 (catalase) displayed later responses and reached their peak expression levels at 6 h. Cp64 (no annotation) exhibited minor changes in its transcript level.

**Figure 7:**
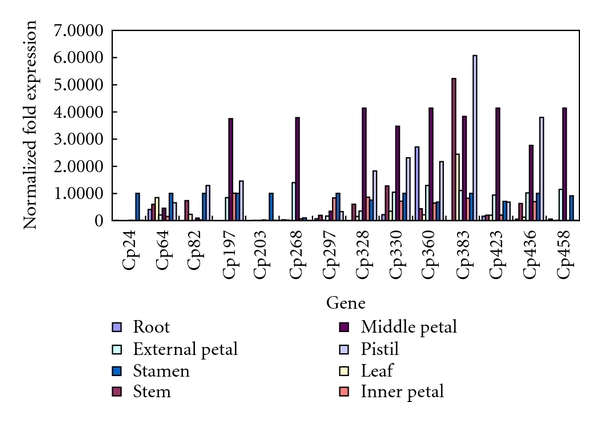
Expression analysis of 14 genes in different tissues of *C. praecox*.

Real-time RT-PCR was also applied to validate the expression patterns of 11 genes related to floral development (Cp197, Cp203, Cp268, Cp297, Cp328, Cp330, Cp360, Cp383, Cp423, Cp436, and Cp458), Cp24, Cp64, and Cp82 (Figure 7). The expression patterns of these 14 genes were analyzed in roots, stems, leaves, outer tepals, middle tepals, inner tepals, stamina, and pistils. The results showed clear differences in their expression. The genes related to floral development, except for Cp203 (LLP-B3 protein) and Cp297 (SRG1-like protein), increased by more than twofold in middle tepals. Cp24 and Cp203 presented an active expression in stamina but showed a very slight accumulation in other tissues. Cp268 (caffeoyl-CoA *O*-methyl-transferase-like protein) and Cp458 (MADS box protein 9) presented a peak in middle tepals but were only slightly or not expressed at all in roots, stems, leaves, and pistils. Cp197 (AGL9.2) was higher in all reproductive organs and presented its peak in middle tepals but was not detected in roots, stems, and leaves. The expression of Cp328 (STYLOSA protein) and that of Cp383 (secondary cell wall-related glycosyltransferase family 47) were not detected in roots; moreover, Cp328 increased by more than fourfold in middle tepals, whereas Cp383 did in pistils and stems. The accumulation of Cp82 (dormancy-related protein) transcripts was higher in stems, leaves, stamina, and pistils. The other genes expressed in all the detected tissues, but Cp64 and Cp297 (SRG1-like protein) presented lower levels of expression. Cp360 (peroxisomal fatty acid *β*-oxidation multifunctional protein AIM1) increased by more than twofold in roots, middle tepals, and pistils.

**Figure 8:**
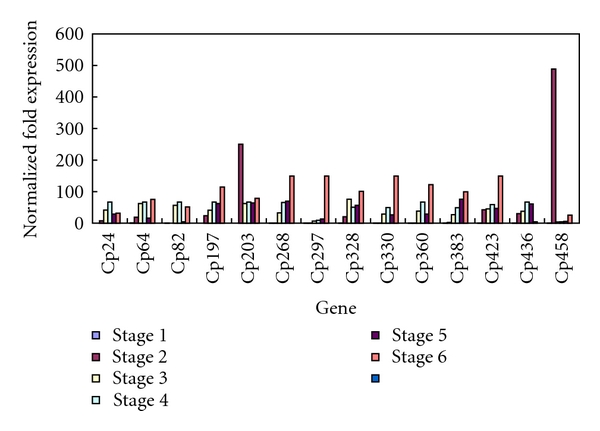
Expression analysis of 14 genes during *C. praecox* flowering.

Quantitative real-time PCR methods were used to validate the transcript levels of the 14 genes further during the blooming process in *C. praecox* (Figure 8). The results showed that all these genes were not detected or very slightly expressed in Stage 1 and that Cp64, Cp82, Cp268, Cp297, Cp330, Cp360, and Cp383 had almost no accumulation in Stage 2. The transcript accumulations of Cp203 and Cp458 were sharply elevated to the highest level in Stage 2 but dramatically decreased at the subsequent stages of floral development. The expressions of Cp24, Cp82, and Cp436 presented a peak in Stage 4. The transcript accumulations of the other 9 genes increased during the six developmental stages and reached their peak in Stage 6. The expression of Cp297(SRG1-like protein) was significantly high in Stage 6 but very low in the other stages, and it was associated with flower senescence. The SRG1 gene is reportedly expressed in senescing organs of *Arabidopsis* plants [36].

The current study found few references about bud dormancy in *C. praecox*. The expression pattern of Cp82 was very attractive. However, the results indicate that Cp82 is not certainly related to flower-bud dormancy. The dormancy-related protein CAA93825.1, which matched Cp82 (*E*-value = 1e^−15^), has been reported to play a role in dormancy breaking and in the germination of *Trollius ledebourii* seeds [37]. These data warrant further research into the relativity of Cp82 to dormancy breaking and germination in *C. praecox* seeds.

## 4. Conclusions

A cDNA library was constructed to generate an EST collection from *C. praecox*, thereby providing a preliminary view into the genomic properties of this species. This collection of high-quality ESTs represents the first EST data set for *C. praecox*. Eight hundred sixty-seven valid EST sequences were generated, and 479 unigenes were assembled, among which 266 unigenes (55.53%) were identified according to their significant similarities with proteins of known functions. The EST sequences have been deposited in GenBank under accession numbers DW222667 to DW223533. Stress response genes and floral development-related genes were also identified. This study evaluated the expression patterns of 23 genes, including 2 novel ones, using real-time RT-PCR. Further investigations in this direction would help in the discovery of promising candidates with a key role in the development of stress tolerance for woody plants to bloom.

## Figures and Tables

**Table 1:** Forward (F) and reverse (R) primers used in real-time PCR.

Gene code	Annotation (sequence homology)	Primer sequence (5′→3′)	Tm (°C) a	Cycles	Product length
Cp88	Membrane channel protein	F: ATGTGGACTTTTTGCTGGAGTTTTT	59	40	90
		R: GCAATACAAGCATTTACCCAGTCCT			
Cp173	Low temperature and salt responsive protein	F: CTGACGCTCTTTGGATGGCTACC	59	40	172
		R: ACGAATCAACGGTCACAAACACG			
Cp215	Putative abscisic acid-induced protein	F: TGCTCTTTACTTGCTGGCTCGTCT	59	40	193
		R: TCTCGCCCATTCTCTTCTATGTATTTC			
Cp274	Glutathione S-transferase	F: ATGTGCTAAATCTCATTCCAGTCTTCC	59	40	85
		R: GCCGTGATATCCTGCGAAACTAG			
Cp359	1,4-Alpha-glucan-maltohydrolase	F: TATTATCTGGCAAGGTTCCACTCGTT	59	40	86
		R: TGTGTTGTAGAACCCAGCAGTCAGC			
Cp364	Cold acclimation Protein WCOR413-like protein beta form	F: TCCAATAAGCCGAATCAAAATACAGT	59	40	84
		R: GTCTGTCTTCATCGCCAAATACTCC			
Cp375	3-Oxoacyl-[acyl-carrier-protein] synthase	F: GTAACTGCGCCGAAACGAGAAAAAG	59	40	78
		R: CCCAAAGACAGAAACCAGCCCC			
Cp440	Putative multiple stress-responsive zinc-finger protein	F: GTTTCCCGATTTTGTGTGGCG	59	40	183
		R: AGTTGTTGATGCAGAGAATTGGTCCT			
Cp465	Catalase	F: CATCGTTCGCTTCTCTACCGTTATT	59	40	118
		R: TGTTTCCGACCAGATCAAAGTTACC			
Cp197	AGL9.2	F: TGAGAATAAGATAAATCGGCAGGTGAC	59	40	128
		R: TTTTCCTCTGCTGGAGAAGACAACG			
Cp203	LLP-B3 protein	F: GCGATGTGAAACTTGTCAGTAGCCC	59	40	171
		R: AGTTTGGCACAATCAGGACGAGGTT			
Cp268	Caffeoyl-CoA *O*-methyl-transferase-like protein	F: AACACAAATGGAGAAGAGAAAACCC	59	40	193
		R: CATCGGCAGAGGTCGTCATAAGA			
Cp297	SRG1-like protein	F: TAAGAACACCGTCCCATCTCGCTAC	59	40	143
		R: GAGTGCAGTCTCTCCAATTCATCAG			
Cp328	STYLOSA protein	F: GGGGAACCAACAAGAACAACAGAT	59	40	161
		R: GCCCAGTGGCACCATTTAGAAGAT			
Cp330	RAC-like G-protein Rac1	F: ACCGCTTCTACCGCCTTTTCTCC	59	40	166
		R: AGGTCTTTCCAACAGCACCATCTCC			
Cp360	Peroxisomal fatty acid betao-xidation multifunctional protein AIM1	F: TATGCTGGTGTTTTGAAAGAGGCG	59	40	193
		R: CTATGCCAGACCCCATCAAACCTC			
Cp383	Secondary cell wall-related glycosyltransferase family 47	F: CAGGCGACACCCCCTCCTCTAAC	59	40	150
		R: TCAAGGCATCAGAGTTACGCACAAAG			
Cp423	Putative polyketide synthase	F: GAGTTCAGACACTGTATGCCCTTGG	59	40	165
		R: TTGCTTGTAAAATTCGTCCTTCGTT			
Cp436	Allergen-like protein BRSn20	F: CTCTTATCGCTCTCTGCTTCCTTCC	59	40	176
		R: GTTGCTGTTAACGTCTCTGCATTCC			
Cp458	MADS-box protein 9	F: TGGTTGAGTATGATGTAGAGAAGCGAC	59	40	98
		R: ACCCATCATCTGAAGGTGTGCTATT			
Cp82	Dormancy related protein	F: TGGAATAGAAAGAGCAAATACAGCG	59	40	172
		R: TACAAAAGGCTCAATGGCGTCC			
Cp24	No hits	F: GCAGTTTACATACTACGGGAGAGGCT	59	40	106
		R: CGGCTTACGGAATCGTCATCAC			
Cp64	Putative protein	F: CCAATCACTCTCCCTGAGGATGTAT	59	40	159
		R: TTCACCGACTCCTTGTTCTTTTAGC			
Internal control	Actin	F: GTTATGGTTGGGATGGGACAGAAAG	59	40	199
		R: GGGCTTCAGTAAGGAAACAGGA			
Internal control	Tublin	F: TAGTGACAAGACAGTAGGTGGAGGT	59	40	139
		R: GTAGGTTCCAGTCCTCACTTCATC			

^a^Annealing temperature.

**Table 2:** Overview of *C. praecox* flowers ESTs.

Items	Number
Total sequenced cDNA	896
Total number of ESTs analyzed	867
Total reading valid length (bp)	584,201
Average EST length (bp)	673.8
Unigenes	479
Contigs	94
Singletons	385
Redundancy	44.8

**Table 3:** The 15 most abundant ESTs in the *C. praecox *flowers cDNA library.

*Gene code *	*Length(bp)*	*Putative function*	*E-value *	*Number of ESTs*
Cp1	1319	lipid transfer protein	1.00*E* − 34	77
Cp2	2124	adenine nucleotide translocator	9.00*E* − 80	47
Cp4	1024	lipid transfer protein	2.00*E* − 35	37
Cp6	1144	hypothetical protein	1.00*E* − 34	19
Cp7	710	seed specific protein Bn15D18B	4.00*E* − 19	18
Cp20	896	mannose specific lectin	5.00*E* − 28	17
Cp16	593	putative LEA III protein isoform 2	6.00*E* − 10	10
Cp10	900	glutathione S-transferase GST 22	4.00*E* − 80	8
Cp11	928	palmitoyl-acyl carrier protein thioesterase	7.00*E* − 22	8
Cp14	1531	hypothetical protein	2.00*E* − 79	8
Cp17	717	stearoyl acyl carrier protein desaturase	2.00*E* − 31	7
Cp26	1074	aquaporin	8.00*E* − 97	7
Cp23	696	S28 ribosomal protein	4.00*E* − 20	6
Cp24	574	No hits	NULL	6
Cp27	652	proline-rich protein	1.00*E* − 30	6

Total	14882	15		281

**Table 4:** Statistics of organism origin of matched-function homologs.

Organism name	No. of unigenes	Percentage (%)^a^
*Arabidopsis thaliana*	132	32.6%
*Oryza sativa (japonica cultivar-group)*	97	24.0%
*Nicotiana tabacum*	17	4.2%
*Glycine max*	8	2.0%
*Lycopersicon esculentum*	7	1.7%
*Gossypium hirsutum*	6	1.5%
*Cucumis sativus*	5	1.2%
*Capsicum annuum*	5	1.2%
*Pisum sativum*	4	1.0%
*Solanum tuberosum*	4	1.0%
*Petunia x hybrida*	4	1.0%
*Nicotiana attenuata*	3	0.7%
*Helianthus annuus*	3	0.7%
*Beta vulgaris*	3	0.7%
*Calycanthus floridus var. glaucus*	3	0.7%
*Ipomoea batatas*	3	0.7%
*Hyacinthus orientalis*	3	0.7%
*Persea americana*	3	0.7%
*Ricinus communis*	3	0.7%
*Lily mottle virus*	3	0.7%
*Medicago sativa*	2	0.5%
*Zea mays*	2	0.5%
*Cicer arietinum*	2	0.5%
*Triticum aestivum*	2	0.5%
*Hevea brasiliensis*	2	0.5%
*Asparagus officinalis*	2	0.5%
*Panax ginseng*	2	0.5%
*Malus x domestica*	2	0.5%
*Elaeis oleifera*	2	0.5%
*Pinus taeda*	2	0.5%
*Spinacia oleracea*	2	0.5%
The other 67 organisms	1	16.5%

^a^Proportion from unigenes with BLAST hits.

**Table 5:** Sequences related to stress and defense.

Gene code	Putative function	*E*-value	Matches no.	Number of ESTs
Cp465	catalase	1.00*E* − 148	BAC79443.1	1
Cp346	transporter	1.00*E* − 138	AAP13421.1	2
Cp382	peroxidase	1.00*E* − 125	AAK52084.1	1
Cp374	GMPase	1.00*E* − 122	AAT58365.1	1
Cp333	putative aldolase	1.00*E* − 122	AAM64281.1	1
Cp397	chitinase	1.00*E* − 105	AAF04454.1	1
Cp452	cysteine synthase	3.00*E* − 99	BAA05965.1	1
Cp314	tryptophan synthase beta chain	2.00*E* − 94	AAN15574.1	1
Cp357	Stromal cell-derived factor 2-like protein	2.00*E* − 93	XP_481233.1	1
Cp191	glutathione S-transferase GST 23	5.00*E* − 92	AAG34813.1	2
Cp25	dehydroascorbate reductase	2.00*E* − 89	AAL71857.1	3
Cp438	ras-related protein RAB8-3	9.00*E* − 89	BAB84324.1	1
Cp459	GTP-binding protein	1.00*E* − 86	AAN31076.1	1
Cp340	putative 3-Hydroxyisobutyryl-coenzyme A hydrolase	9.00*E* − 84	AAN41356.1	1
Cp379	cell-autonomous heat shock cognate protein 70	1.00*E* − 82	gAAN86276.1	1
Cp39	auxin-binding protein	3.00*E* − 80	AAB51240.1	4
Cp10	glutathione S-transferase GST 22	4.00*E* − 80	AAG34812.1	8
Cp304	putative 2-Nitropropane dioxygenase	2.00*E* − 79	AAL34288.1	3
Cp21	glutathione S-transferase GST 22	2.00*E* − 78	AAG34812.1	3
Cp311	GTP-binding protein	6.00*E* − 73	AAM12880.1	1
Cp353	patatin-like protein	1.00*E* − 73	CAB16788.1	2
Cp274	glutathione S-transferase	1.00*E* − 72	AAF61392.1	1
Cp359	1,4-Alpha-glucan-maltohydrolase	1.00*E* − 71	CAH60892.1	1
Cp59	BTF3b-like transcription factor	2.00*E* − 68	AAT67244.1	3
Cp76	NDKB_FLABI Nucleoside diphosphate kinase B	2.00*E* − 63	P47920	1
Cp166	putative tropinone reductase	6.00*E* − 59	AAM62552.1	1
Cp313	putative glutathione S-transferase T3	5.00*E* − 56	AAG16758.1	1
Cp156	protolysis and peptidolysis	6.00*E* − 56	CAA50022.1	1
Cp120	putative pyruvate dehydrogenase E1 alpha subunit	8.00*E* − 56	XP_467697.1	1
Cp80	sadtomato protein	7.00*E* − 55	AAS77347.1	1
Cp440	putative multiple stress-responsive zinc-finger protein	6.00*E* − 54	BAD35553.1	1
Cp175	epoxide hydrolase	6.00*E* − 50	AAB02006.1	1
Cp364	cold acclimation protein WCOR413-like protein beta form	4.00*E* − 49	AAG13394.1	1
Cp471	pathogenesis-related protein 4B	1.00*E* − 48	eCAA41438.1	1
Cp145	Thioredoxin	4.00*E* − 44	CAA77847.1	1
Cp406	auxin-responsive family protein	1.00*E* − 43	NP_565113.1	1
Cp38	basic PR-1 protein precursor	3.00*E* − 43	AAU20808.1	4
Cp215	putative abscisic acid-induced protein	4.00*E* − 41	BAD37454.1	1
Cp66	GLRX_RICCO Glutaredoxin	5.00*E* − 40	sp|*P*55143|	2
Cp235	histone H2A	5.00*E* − 38	CAA07234.1	1
Cp111	topoisomerase 6 subunit B	5.00*E* − 37	CAC24690.1	1
Cp232	cysteine proteinase inhibitor-like protein	2.00*E* − 36	BAB03156.1	2
Cp220	glyoxalase I family protein	2.00*E* − 35	NP_973579.1	2
Cp214	acyl-CoA-binding protein	3.00*E* − 35	CAA70200.1	1
Cp27	proline-rich protein	1.00*E* − 30	AAF78903.1	6
Cp20	lectin	5.00*E* − 28	AAD45250.1	17
Cp134	metallothionein-like protein	6.00*E*−27	CAB52585.1	1
Cp127	Gip1-like protein	2.00*E* − 24	CAD10106.1	1
Cp296	bZIP transcription factor	2.00*E* − 24	AAN61914.1	2
Cp375	3-Oxoacyl-[acyl-carrier-protein] synthase	2.00*E* − 23	AAC78479.1	1
Cp104	metallothionein-like protein type 2	4.00*E* − 18	CAB77242.1	2
Cp372	allyl alcohol dehydrogenase	5.00*E* − 17	BAA89423.1	1
Cp36	metallothionein-like protein type 2	1.00*E* − 13	AAV97748.1	5
Cp173	low temperature and salt responsive protein	1.00*E* − 12	BAC23051.1	1
Cp472	cystatin	2.00*E* − 07	AAO18638.1	1
Cp88	membrane channel protein	3.00*E* − 06	CAB83138.1	2
Also related to development				
Cp391	actin	1.00*E* − 166	AAN40685.1	1
Cp402	xyloglucan endotransglycosylase precursor	1.00*E* − 110	AAC09388.1	1
Cp26	aquaporin	8.00*E* − 97	CAE53881.1	7
Cp381	4-Coumarate-CoA ligase-like protein	2.00*E* − 89	AAP03021.1	1
Cp461	glyceraldehyde-3-phosphate dehydrogenase	1.00*E* − 86	CAC88118.1	1
Cp291	3-Ketoacyl-CoA thiolase	6.00*E* − 84	CAA47926.1	1
Cp378	probable ubiquitin-conjugating enzyme E2	2.00*E* − 83	AAC32141.1	1
Cp400	auxin response factor 4	5.00*E* − 81	BAD19064.1	1
Cp288	expansin	4.00*E* − 80	AAO15998.1	2
Cp226	calmodulin	3.00*E* − 79	AAB68399.1	4
Cp323	putative calmodulin	3.00*E* − 73	CAC84563.1	2
Cp298	putative adrenal gland protein AD-004	2.00*E* − 65	XP_477670.1	1
Cp326	UDP-glucose:salicylic acid glucosyltransferase	2.00*E* − 65	AAF61647.1	1
Cp58	actin-depolymerizing factor 1	1.00*E* − 63	AAK72617.1	2
Cp167	ABC transporter	4.00*E* − 60	AAP80385.1	1
Cp286	putative CCR4-associated factor	4.00*E* − 60	AAN13153.1	1
Cp368	MYB8 protein	1.00*E* − 60	CAD87008.1	1
Cp324	actin depolymerizing factor	2.00*E* − 59	AAD23407.1	1
Cp450	TAF9	1.00*E* − 58	AAR28026.1	1
Cp405	myb family transcription factor-like	2.00*E* − 52	BAD29385.1	1
Cp77	putative actin-depolymerizing factor 1	4.00*E* − 48	XP_475079.1	1
Cp121	TAF10	3.00*E* − 41	AAR28030.1	1
Cp347	putative transcription factor	2.00*E* − 38	XP_479103.11	2
Cp4	lipid-transfer protein	2.00*E* − 35	AAS13435.1	37
Cp1	lipid transfer protein	1.00*E* − 34	CAA63340.1	77
Cp275	lipid transfer protein precursor	4.00*E* − 30	AAL27855.1	2
Cp9	lipid transfer protein 1 precursor	3.00*E* − 25	AAT45202.1	1
Cp216	lipid-transfer protein	3.00*E* − 24	NP_915960.1	4
Cp90	zinc finger homeobox family protein	3.00*E* − 22	NP_565088.1	1
Cp41	lipid transfer protein	3.00*E* − 18	BAC77694.1	1
Cp13	lipid transfer protein isoform 4	1.00*E* − 16	AAO33394.1	1
Cp338	polyprotein	2.00*E* − 16	CAD92110.1	1
Cp82	dormancy related protein, putative	6.00*E* − 15	AAG51119.1	1
Cp8	lipid-transfer protein	7.00*E* − 14	AAS13435.1	1
Cp141	lipid-transfer protein	4.00*E* − 13	AAS13435.1	1
Cp211	neutral/alkaline invertase 1	2.00*E* − 12	AAV28809.1	1
Cp451	putative MYB family transcription factor	8.00*E* − 11	AAD23043.1	1
Cp34	Lea5 protein	3.00*E* − 10	CAA86851.1	4
Cp16	putative LEA III protein isoform 2	6.00*E* − 10	CAC39110.1	10

**Table 6:** Sequences related to floral development.

*Gene code*	*Putative function*	*E-value*	*Matches no.*	*Number of ESTs*
Cp360	Peroxisomal fatty acid beta-oxidation multifunctional protein AIM1	1.00*E* − 108	XP_464920.1	1
Cp268	caffeoyl-CoA *O*-methyl-transferase-like protein	1.00*E* − 107	CAB80122.1	2
Cp237	MADS box protein	1.00*E* − 106	AAQ83835.1	2
Cp116	MADS box transcription factor AP3-1	2.00*E* − 95	AAF73928.1	1
Cp330	RAC-like G-protein Rac1	1.00*E* − 93	AAD47828.2	1
Cp217	beta-D-glucosidase	2.00*E* − 92	AAQ17461.1	1
Cp413	leucine-rich repeat transmembrane protein kinase	6.00*E* − 86	NP_192248.2	1
Cp408	isoflavone reductase related protein	3.00*E* − 85	AAC24001.1	1
Cp423	putative polyketide synthase	8.00*E* − 76	NP_919053.1	1
Cp335	AGL 6	3.00*E* − 73	AAY25580.1	2
Cp395	isopentenyl pyrophosphate:dimethyllallyl pyrophosphate isomerase	2.00*E* − 70	BAB09611.1	1
Cp297	SRG1-like protein	1.00*E* − 61	CAB81342.1	3
Cp383	secondary cell wall-related glycosyltransferase family 47	3.00*E* − 49	AAX33321.1	1
Cp197	AGL9.2	4.00*E* − 42	AAX15924.1	1
Cp436	allergen-like protein BRSn20	1.00*E* − 37	AAM62935.1	1
Cp407	caffeate *O*-methyltransferase	8.00*E* − 23	AAV36331.1	1
Cp203	LLP-B3 protein	1.00*E* − 22	AAN76546.1	1
Cp328	STYLOSA protein	1.00*E* − 15	CAF18245.1	1
Cp458	MADS-box protein 9	9.00*E* − 15	AAQ72497.1	1
